# Overexpression of Id1 in transgenic mice promotes mammary basal stem cell activity and breast tumorigenesis

**DOI:** 10.18632/oncotarget.3640

**Published:** 2015-04-16

**Authors:** Dong-Hui Shin, Ji-Hye Park, Jeong-Yeon Lee, Hee-Young Won, Ki-Seok Jang, Kyueng-Whan Min, Si-Hyong Jang, Jong-Kyu Woo, Seung Hyun Oh, Gu Kong

**Affiliations:** ^1^ Department of Pathology, College of Medicine, Hanyang University, Seoul, Republic of Korea; ^2^ Institute for Bioengineering and Biopharmaceutical Research (IBBR), Hanyang University, Seoul, Republic of Korea; ^3^ College of Pharmacy, Gachon University, Incheon, Republic of Korea

**Keywords:** basal-like breast cancer, cancer stem cell, Id1, mammary stem cell, c-Myc

## Abstract

Inhibitor of differentiation/DNA binding (Id)1 is a crucial regulator of mammary development and breast cancer progression. However, its effect on stemness and tumorigenesis in mammary epithelial cells remains undefined. Herein, we demonstrate that Id1 induces mammary tumorigenesis by increasing normal and malignant mammary stem cell (MaSC) activities in transgenic mice. MaSC-enriched basal cell expansion and increased self-renewal and *in vivo* regenerative capacity of MaSCs are observed in the mammary glands of MMTV-Id1 transgenic mice. Furthermore, MMTV-Id1 mice develop ductal hyperplasia and mammary tumors with highly expressed basal markers. Id1 also increases breast cancer stem cell (CSC) population and activity in human breast cancer lines. Moreover, the effects of Id1 on normal and malignant stem cell activities are mediated by the Wnt/c-Myc pathway. Collectively, these findings provide *in vivo* genetic evidence of Id1 functions as an oncogene in breast cancer and indicate that Id1 regulates mammary basal stem cells by activating the Wnt/c-Myc pathway, thereby contributing to breast tumor development.

## INTRODUCTION

Inhibitor of differentiation and DNA binding (Id)1, a member of the Id protein family that functions as a dominant–negative regulator of basic HLH transcription factors, has been known to play a crucial role in mammary epithelial cells and breast carcinomas by mediating diverse cellular functions, including differentiation, proliferation, and invasion and metastasis [1–5]. Clinically, higher Id1 level is positively associated with poor patient outcome, high tumor stage, aggressiveness and metastasis in human breast cancer [6–9]. We have also provided evidence that Id1 has an oncogenic role in malignant mammary epithelial cells by promoting cell growth, resistance to antitumor therapy, and angiogenesis [10–12]. Furthermore, our previous study showed that constitutive overexpression of Id1 is critical for mammary gland development through induction of precocity and alveologenesis, and delayed involution in MMTV-transgenic mice model via regulating Wnt signaling and Bcl-2 [13]. However, although the potential significant role of Id1 in mammary gland development, whether Id1 is involved in the regulation of mammary stem cell (MaSC) maintenance and differentiation remains unclear.

MaSCs, a subpopulation of basal cells with self-renewal and multi-lineage differentiation abilities, have been identified in mammary glands [14]. These cells can generate normal-looking mammary glands and ducts, suggesting that they play an important role in mammary gland development [15]. Moreover, the recent identification of different progenitor populations in the mouse mammary epithelium suggests that MaSCs can give rise to committed progenitor cells for either the myoepithelial or luminal epithelial lineage and regulate organization of the mammary epithelium in a hierarchical manner [16]. Several mammary stem/progenitor regulators including Notch, Gata-3, and BRCA1 specify luminal cell fate [17–19], while some regulators such as Wnt signaling are involved in maintenance of basal lineage in the mammary gland [16]. Furthermore, growing evidence has shown that abnormality of mammary stem and progenitor cells by deregulation of these factors affecting the mammary hierarchy causes the development of different breast tumor subtypes including luminal and basal breast cancers [20, 21], implying the potential role of mammary stem/progenitor cells in regulating tumorigenesis of the mammary gland, as well as normal development.

Despite the importance of Id1 in mammary development and breast cancer progression, its effect on stemness and differentiation in mammary epithelial cells and its association with mammary tumorigenesis remain to be further elucidated. Herein, using MMTV-Id1 transgenic mice, we identified that Id1 plays a crucial role in the regulation of normal and malignant mammary stem cells by activating the Wnt/c-Myc pathway, thereby contributing to basal marker-positive breast tumor development.

## RESULTS

### Id1 expands the MaSC population and enhances self-renewal activity

**Figure 1:**
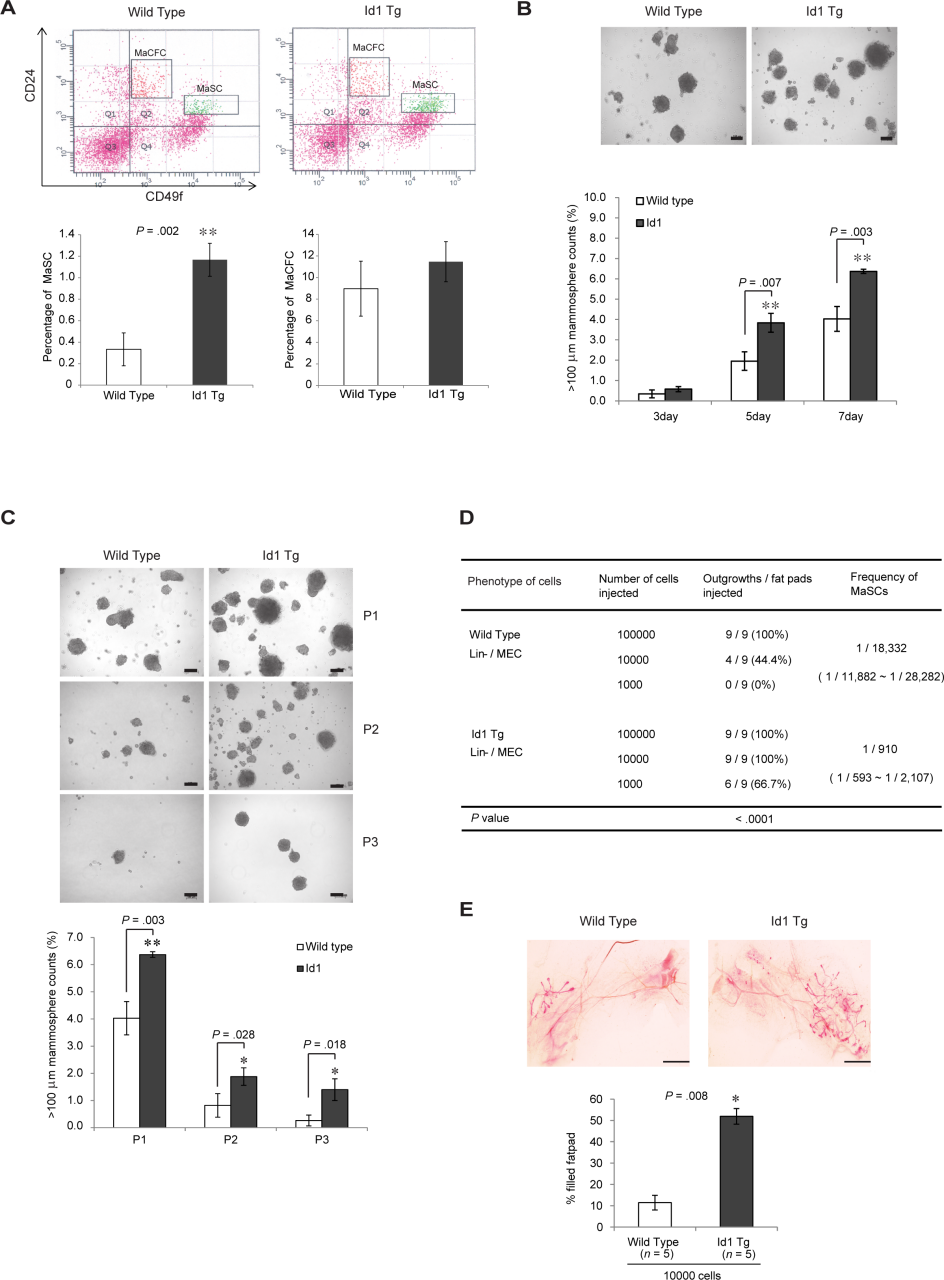
Id1 increases the mammary stem cell population and self-renewal activity **A.** FACS analysis of CD24 and CD49f expression in the Lineage (Lin)-negative population of MECs (top). The Lin^−^/CD24^med^/CD49f^high^ MaSC and Lin^−^/CD24^high^/CD49f^+^ MaCFC percentages are shown in the bar graph (bottom panel). **B.** Formation of mammospheres from MECs from 12-week-old virgin wild-type and MMTV-Id1 mice. Numbers of spheres (diameter > 100 μm) formed were counted. **C.** The first forming spheres (P1) were re-plated under identical conditions to generate second (P2) and third (P3) passage mammospheres. Numbers of spheres (>100 μm) formed were counted. Scale bars in B–C = 100 μm. Error bars in A–C represent means ± SD of triplicate measurements. *P < .05, **P < .01, ***P < .001 vs. control (Student *t* test). **D.** Analysis of MaSC frequency in wild-type and MMTV-Id1 mice (*n* = 9). Gland-reconstituting activities were measured by limiting dilution cell transplantation experiments. *P* < .0001 based on Poisson distribution. **E.** Whole-mount staining of outgrowth from transplanted MECs from wild-type and MMTV-Id1 mice. The histogram represents the percentage of the fat pad filled by reconstituted mammary ductal trees. Results are shown as the means ± SEM (*n* = 5 independent experiments; ***P = .008, Mann-Whitney *U* test).

To investigate the potential role of Id1 in the regulation of normal mammary stem/progenitor cells in mammary glands, we initially examined the mouse MaSC and progenitor populations by fluorescence-activated cell sorting (FACS) analysis using the cell surface phenotype markers CD24, CD29, and CD49f in wild-type and MMTV-Id1 transgenic mice. The CD24^med^/CD49f^high^ MaSC population, also known as mammary repopulating units (MRUs) [14], was expanded in the mammary glands of MMTV-Id1 mice compared with wild-type mice (3.5-fold increase, *P* = 0.002, two-sided *t* test; Figure 1A, top and bottom left). In contrast, no apparent difference was noted in the population of mammary epithelial progenitor cells, also known as MaCFCs, between MMTV-Id1 and wild-type mice (Figure 1A, top and bottom right). We next investigated whether Id1 affected the self-renewal activity of MaSCs. The number of mammospheres was significantly increased in MMTV-Id1 transgenic mice compared with wild-type mice (Figure 1B) and that this increase was maintained on serial passage to tertiary mammospheres (Figure 1C). The functional assay of mammary fat pad repopulation showed extensive mammary epithelial outgrowth in transplants of MECs from MMTV-Id1 mammary glands (*n* = 9, frequency: 1 in 910 cells compared to 1 in 18, 332 cells in MECs from wild-type mice, *P* < 0.0001, Poisson distribution; Figure 1D). Moreover, the outgrowth in MMTV-Id1 mice resulted in a significant increase of the mammary tree ductal size (51.89% of reconstituted mammary ductal trees compared to 11.43% of those in wild type mice; *P* = 0.008, Mann-Whitney *U* test; Figure 1E). Together, these results indicate that Id1 is able to enrich the MaSC population and enhance the self-renewal and repopulation capacity of the stem cells.

### Id1 maintains the MaSC-enriched basal cells, but not the luminal cell lineage

**Figure 2:**
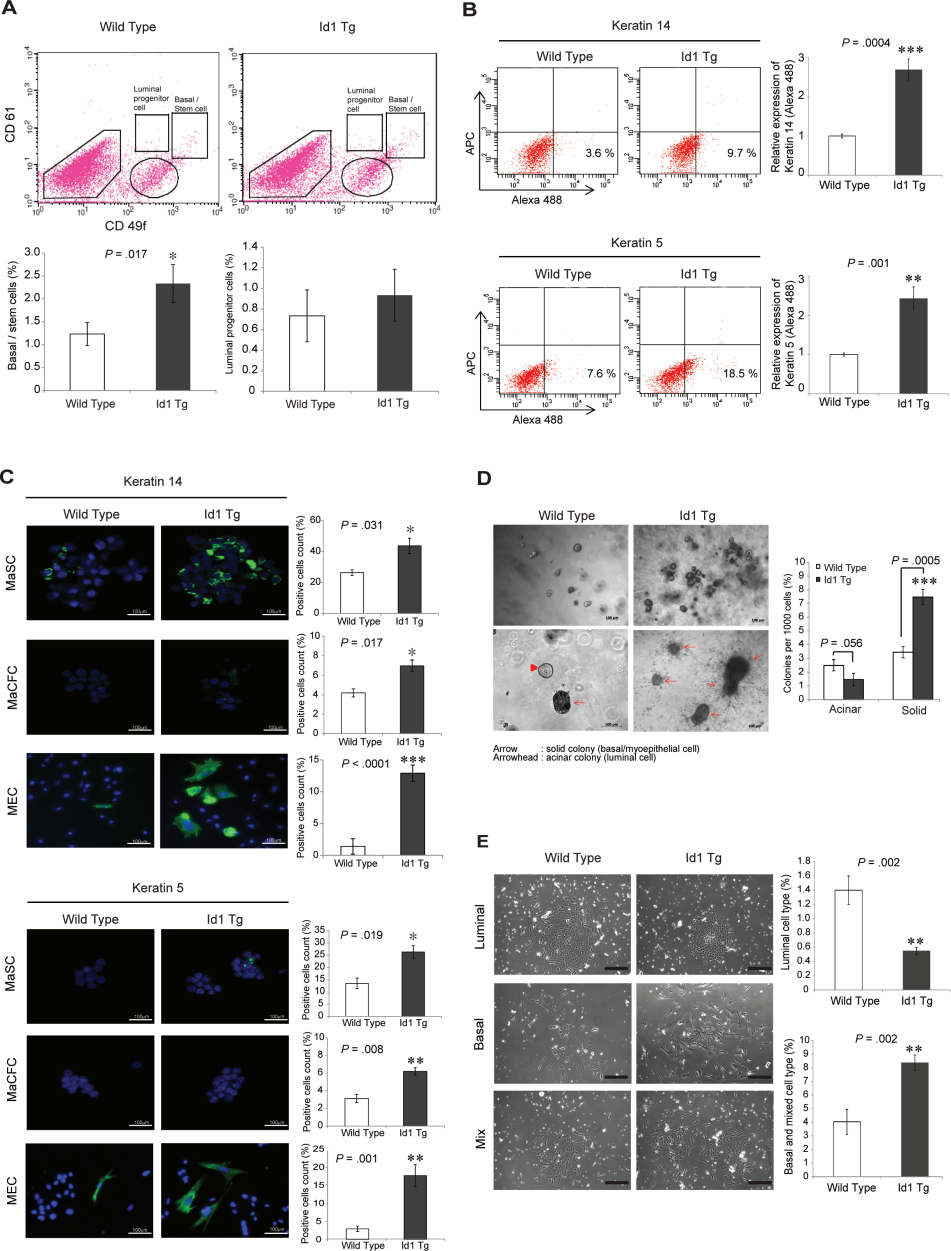
Id1 induces the expansion of the MaSC-enriched basal subpopulation **A.** FACS analysis of CD61 and CD49f expression in the Lin-negative population of MECs. The percentages of Lin^−^/CD61^+^/CD49f^high^ MaSC-enriched basal cells (left panel) and Lin^−^/CD61^+^/CD49f^low^ luminal progenitor cells (right panel) are shown in a bar graph. **B.** Expression of keratin 5 and keratin 14 in MECs as determined using flow cytometry. The percentage of the basal marker-expressing cells (Alexa Fluor 488 positive; lower right quadrant of the dot plots) was indicated as a bar graph. The APC channel was used as a negative control to determine background fluorescence. **C.** Expression of keratin 5 and keratin 14 in MaSCs, MaCFCs, and MECs was measured with immunocytochemistry and is shown as bar graphs. **D.** Epithelial progenitor colonies from single-cell suspensions of primary mammospheres in MECs of wild-type and MMTV-Id1 mice in three-dimensional (3-D) culture (left panels, top and bottom, low and high power magnification images showing the incidence of colony formation and types of colonies (acinar *vs*. solid), respectively). The histogram shows percentages of the different types of progenitor colonies (right panel). **E.** *In vitro* epithelial colony-forming assay with single-cell suspensions of primary mammospheres in MECs of wild-type and MMTV-Id1 mice (left panel). Histograms represent the percentages of luminal colonies (top-right panel), and basal and mixed colonies (bottom-right panel). Scale bar in C–E. = 100 μm. Results in A–E. are shown as the means ± SD of three independent experiments. *P < .05, **P < .01, ***P < .001 vs. controls (Student *t* test).

To examine whether Id1 is involved in determining the mammary epithelial lineage as a regulator of MaSCs, we next characterized the distinct mammary cell subpopulations in wild-type and MMTV-Id1 mammary glands. The FACS analysis with CD49f and CD61 markers as described previously [17, 22] showed that the Lineage (Lin)^−^/CD61^+^/CD49f^high^ MaSC-enriched basal cell population was augmented in MMTV-Id1 mammary glands compared with wild-type glands (Figure 2A, top and bottom left panels). In contrast, no significant differences were observed in the Lin^−^/CD61^+^/CD49f^low^ luminal progenitor and Lin^−^/CD61^−^/CD49f^low^ differentiated luminal cell populations between MMTV-Id1 and wild-type glands (Figure 2A, top and bottom right panels). Consistent with these observations, the MECs from MMTV-Id1 mice expressed high levels of the basal lineage markers keratin 5 (K5) and K14 as assessed by FACS analysis (Figure 2B). We also examined the expression of these markers in MaSC and MaCFC populations, as well as MECs from MMTV-Id1 and wild-type mice, using immunofluorescence staining. The basal markers were highly expressed in the MaSC-enriched basal population isolated from MMTV-Id1 mice (Figure 2C, top panel). Although the MaCFC subpopulation showed low levels of K5 and K14 expression, their expression was slightly increased in MaCFCs from MMTV-Id1 glands (Figure 2C, middle panel). MaSC-enriched basal cells were reported to form solid organoids, while luminal progenitors formed acinar colonies in three-dimensional (3–D) culture [14, 22]. Consistently, the single cells from dissociated primary mammospheres in MMTV-Id1 mammary glands preferentially formed solid organoids (Figure 2D). Furthermore, using an *in vitro* epithelial colony-forming assay that distinguishes among luminal, myoepithelial, and mixed colonies [23, 24], we found that MECs of MMTV-Id1 mice formed a twofold greater number of myoepithelial colonies and fewer luminal colonies compared with MECs of wild-type mice, indicating basal cell fate specification by Id1 in mammary glands (Figure 2E). Consistent with these observations, there was no significant difference in the expression of luminal markers, K8, estrogen receptor (ER), and progesterone receptor (PR), between MMTV-Id1 and wild-type mice (Supplementary Figure S1). These findings suggest that Id1 induces expansion of the MaSC-enriched basal subpopulation, but not the luminal cell lineage, in mammary glands.

### Id1 induces ductal hyperplasia and mammary tumors with high expression of basal markers

**Figure 3:**
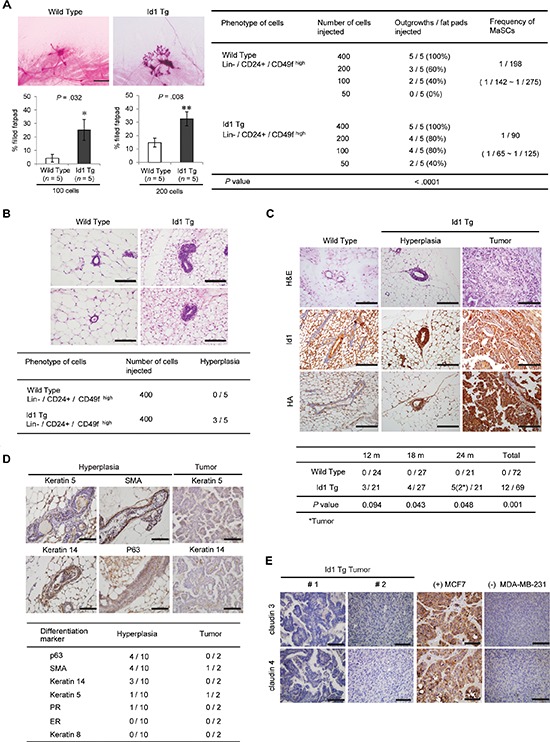
Development of ductal hyperplasia and mammary tumors with positive basal markers in MMTV-Id1 transgenic mice **A.** Reconstituted mammary glands transplanted with Lin^−^/CD24^med^/CD49f^high^ MaSC cells from wild-type and MMTV-Id1 mammary glands. The histogram represents the percentage of reconstituted mammary ductal trees after transplantation of the indicated numbers of cells (left panel). Results are shown as the means ± SEM (*n* = 5 independent experiments); *P < .05, **P < .01 vs. control, Mann-Whitney *U* test). Limiting dilution cell transplantation experiments, with analysis using L-Calc software and Poisson distribution for calculating MaSCs frequency and *P*-value (right panel). **B.** Ductal hyperplasia developed in reconstituted mammary ductal trees derived in MaSCs from Id1 transgenic mice (*n* = 5; H&E, original magnification × 400). Based on the histological analysis, the intraductal epithelial proliferation with multilayered ductal lining was considered as ductal hyperplasia. **C.** Ductal hyperplasia and mammary tumors occurred in wild type (*n* = 72) and MMTV-Id1 transgenic virgin mice (*n* = 69) over 24 months. Expression of the exogenous Id1 transgene in the tumor tissue was detected with Id1 and HA antibodies (top panel). Incidence of ductal hyperplasia and mammary tumors were analyzed statistically by chi-square test (bottom panel). **D.** Immunohistochemical analysis of basal and luminal markers in the 10 cases of hyperplasia and two tumors derived from MMTV-Id1 mice. **E.** Expression of claudin proteins in mammary tumors derived from MMTV-Id1 transgenic mice. Representative images of immunohistochemical staining for claudin 3 and claudin 4 in tumor tissues of MMTV-Id1 mice are shown. The tumor tissues obtained from xenograft mice with MCF7 and MDA-MB-231 cells were used for positive and negative controls of staining claudins, respectively. All experiments were performed in triplicate. Scale bar in A–E. = 100 μm.

Previous studies suggested that increased mammary stem/progenitor cell activity influences breast cancer risk [19, 21, 25]. To determine whether Id1 contributes to mammary tumorigenesis by activating MaSCs, we transplanted CD24^med^/CD49f^high^ cells isolated from the mammary glands of MMTV-Id1 or wild-type mice at a limiting dilution into cleared mammary fat pads. Eight weeks after transplantation, ductal size had increased in mammary fat pads inoculated with MaSCs of MMTV-Id1 mice compared with those inoculated with wild-type MaSCs (*P* = 0.032 and 0.008 for 100 and 200 cells, respectively, Mann-Whitney *U* test; Figure 3A, left panel). Moreover, fewer cells could form ductal structures in transplants of MaSCs from MMTV-Id1 mammary glands compared with those of wild-type MaSCs, indicating that Id1 increases outgrowth potential (*n* = 5, *P* < 0.0001, Poisson distribution; Figure 3A, right panel). Notably, histological analysis showed that ductal hyperplasia with multilayer epithelium was detected 2 months after injection in transplants of MaSCs from MMTV-Id1 mammary glands (Figure 3B). We further examined whether mammary preneoplasia develops in MMTV-Id1 mice after the long-term observation. At 24 months of age, none of the wild-type mice developed a tumor, whereas 10 cases of ductal hyperplasia and two tumors were observed in a total of 69 MMTV-Id1 mice in which constitutive Id1 overexpression retained well (17.39%, *P* = 0.001, chi-square test; Figure 3C). Furthermore, these tumors exhibited ductal carcinomas with marked nuclear pleomorphism and increased mitotic counts. We also found that the ductal hyperplasia and mammary tumors in MMTV-Id1 mice exhibited expression of the basal markers K5, K14, SMA, and p63, implying the generation of breast tumors with basal marker positivity by Id1 (Figure 3D). In addition, these tumors had low expression of claudin 3 and claudin 4, possibly implying the claudin-low subtype of breast tumor (Figure 3E). Taken together, these findings suggest that Id1 induces basal marker-expressing breast tumor by increasing deregulation of mammary basal stem cells, thereby contributing to mammary tumorigenesis.

### Id1 increases the cancer stem cell properties in human breast cancer

**Figure 4:**
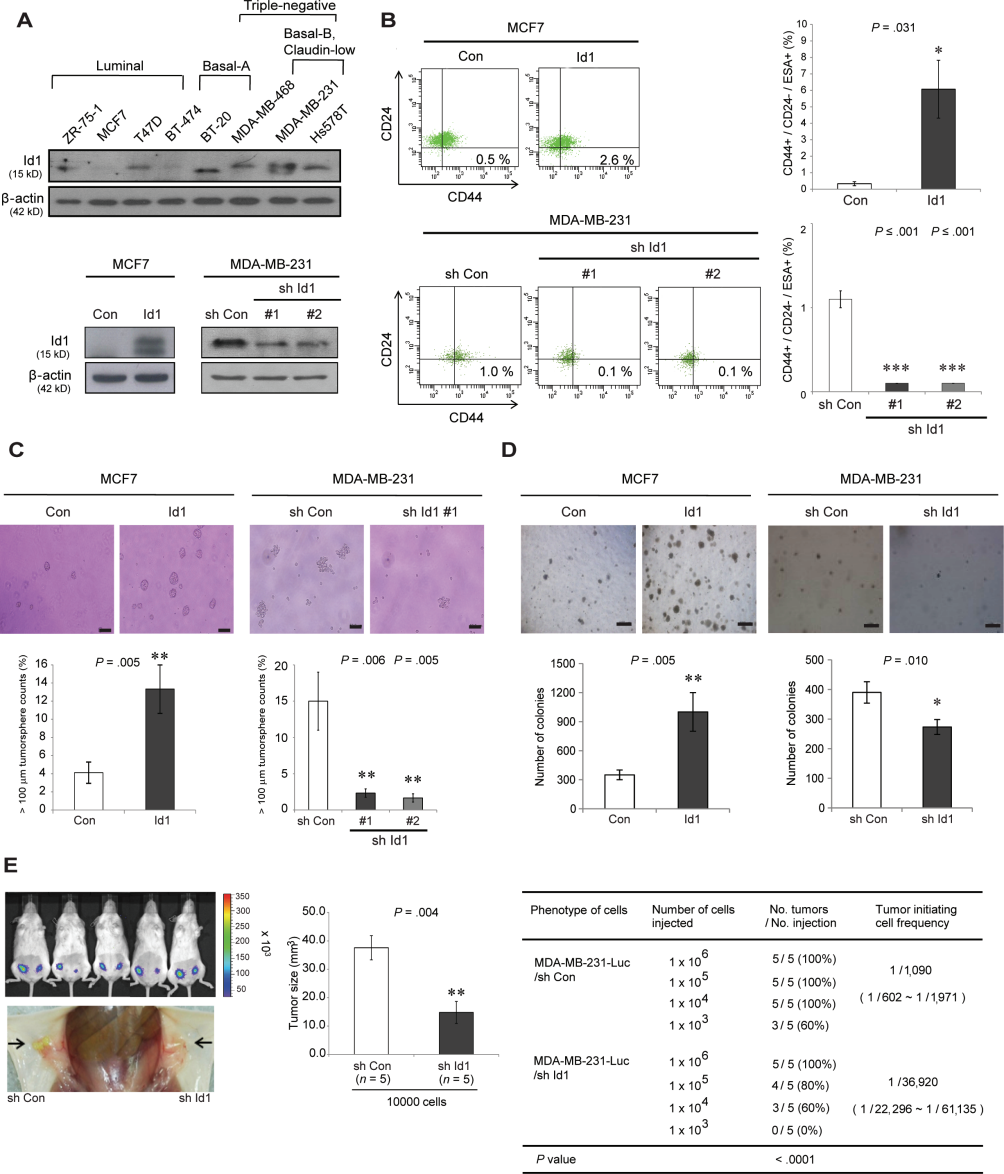
Id1 enhances the stemness of breast cancer stem cells **A.** Cell lysates of indicated human breast cancer cell lines, MCF7 cells stably transfected with an empty vector (Con) or Id1 cDNA (Id1), and MDA-MB-231 cells infected with lentiviruses encoding control shRNA (sh Con) or Id1 shRNAs (sh Id1 #1 and #2) were analyzed for expression of Id1 using Western blot analysis. **B.** FACS analysis of CD44, CD24, and ESA expression in the indicated cell lines (top panel). The histogram shows quantification of ESA^+^/CD44^+^/CD24^−^cells (bottom panel). Data are shown as the means ± SEM (*n* = 3 independent experiments). **C.** Effects of Id1 on tumorsphere formation. Numbers of tumorspheres (diameter > 100 μm) formed were counted. Scale bar = 100 μm. **D.** A soft agar colony formation assay was performed to measure anchorage-independent tumor growth. Numbers of colonies formed (diameter > 50 μm) were counted. Scale bar = 100 μm. Results in C. and D. are shown as the means ± SD of three independent experiments (*P < .05, **P < .01 vs. control, Student *t* test; in B–D. **E.** Limiting dilution assay of Id1 knockdown MDA-MB-231-Luc cells stably expressing control shRNA or Id1 shRNA in NOD/SCID mice (*n* = 5) using orthotopic xenografts. *In vivo* bioluminescence imaging for monitoring tumor formation (left panel) and a bar graph for tumor size of indicated groups (middle panel). Data represent the means ± SEM (**P < .01 vs. control, Student *t* test). Tumor-initiating cell frequency was calculated using L-Calc software and summarized as a table (right; *P* < .0001 based on Poisson distribution).

Because Id1 contributed to the development of ductal hyperplasia and mammary tumors, we investigated whether Id1 could influence CSC activity in human breast cancer cell lines. Consistent with the observation in MMTV-Id1 mice, Id1 was more strongly expressed in basal-type breast cancer cell lines (Figure 4A, top panel). Furthermore, in Id1-overexpressing MCF7 luminal type breast cancer cells, the CD44^+^/CD24^−^/ESA^+^ breast CSC population was enriched, whereas short hairpin RNAs (shRNAs)-mediated Id1 knockdown in MDA-MB-231 basal-like type breast cancer cells reduced the size of this population (Figures 4A, bottom panel, and 4B). The self-renewal of breast CSCs was also enhanced by Id1 in MCF7 cells and inhibited by Id1 knockdown in MDA-MB-231 cells, as assessed by tumorsphere formation assay (Figure 4C). In these cells, the ability for anchorage-independent tumor growth was also positively regulated by Id1 (Figure 4D). Furthermore, the reduction of Id1 expression impaired the *in vivo* tumor-initiating ability of orthotopic xenografts (*n* = 5; frequency: 1/36,920 cells compared to 1/1,090 cells, *P* < 0.0001, Poisson distribution; Figure 4E, right panel), as well as *in vivo* tumor growth (Figure 4E, left and middle panels). These findings suggest that Id1 contributes to mammary tumorigenesis by enhancing normal and malignant mammary stem cell activities.

### Id1 promotes normal and cancer stem cell activities through the Wnt/c-Myc pathway

**Figure 5:**
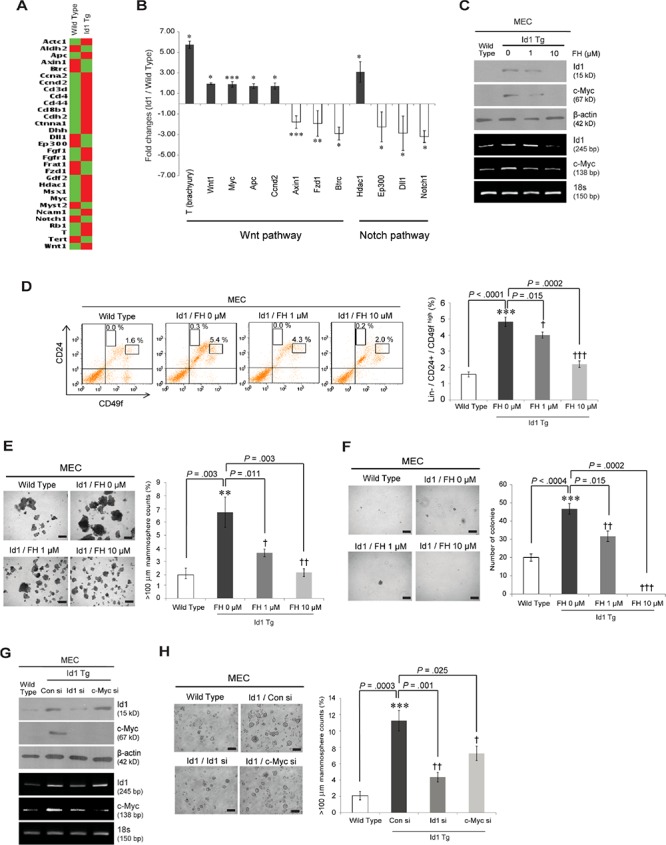
Id1 promotes mammary stem cell activities through the Wnt-mediated c-Myc upregulation **A.** Heat map of the gene expression profiles. A statistically significant > 1.5-fold change in MaSCs from MMTV-Id1 mice compared with those from wild-type mice was detected using Mouse Stem Cell RT^2^ Profiler PCR Arrays. The result is the average value of three independent MaSC samples. **B.** Pathway analysis of Id1 target gene lists from the arrays. Data represent the means ± SD (*n* = 3 independent samples; *P < .05, **P < .01, ***P < .001 vs. control, Student *t* test). **C.** MECs from MMTV-Id1 mice were treated with the indicated dose of the β-catenin/TCF inhibitor FH535 (FH) for 48 h, and the expression levels of Id1 and c-Myc were detected by reverse transcription (RT)-PCR and Western blot analysis. 18s rRNA was used as an internal loading control. **D.** FACS analysis of CD24 and CD49f expression in Lineage (Lin)-negative MECs from MMTV-Id1 mice after treatment of the MECs with FH535. The histogram shows Lin^−^/CD24^med^/CD49f^high^ MaSC percentages (left panel). The bar graph shows the quantification of the result (right panel). **E and F.** Mammosphere-forming E. and soft agar colony-forming F. abilities of MECs from MMTV-Id1 mice after inhibition of Wnt/c-Myc signaling by FH535. **G.** MECs from MMTV-Id1 mice were treated with the indicated siRNAs for 48 h, and the expression levels of Id1 and c-Myc were detected by RT-PCR and Western blot analysis. **H.** Mammosphere-forming ability of MECs from MMTV-Id1 mice treated with Id1 or c-Myc siRNA. All experiments were performed in triplicate. Results in D–F. and H. are shown as the means ± SD (*n* = 3 independent experiments; */^†^*P* < .05; **/^††^*P* < .01; ***/^†††^*P* < .001 vs. the wild type and Id1 controls, respectively, Student *t* test).

To determine the molecular mechanism by which Id1 regulates normal and malignant stem cell activities, the expression profiles of stem cell regulators in MaSCs from MMTV-Id1 and wild-type mice were examined in a stem cell PCR array. Notably, we found that the majority of Id1 target genes are involved in the Wnt pathway (Figures 5A and 5B, and Supplementary Table S1). Such Wnt signaling components, including the Wnt ligand Wnt-1 (1.94-fold, *P* = 0.040, two-sided *t* test) and the Wnt/β-catenin/TCF target genes c-Myc (1.89-fold, *P* < 0.0001, two-sided *t* test) and brachyury (5.74-fold, *P* = 0.034, two-sided *t* test), were upregulated in MaSCs isolated from MMTV-Id1 mice compared with wild-type MaSCs (Figure 5B and Supplementary Table S1). In contrast, luminal progenitor regulator Notch signaling components were downregulated in these cells. Because the canonical Wnt signaling pathway plays an essential role in regulating self-renewal of both normal and cancer stem cells [26–29], we explored whether Id1 enhanced normal and malignant mammary stem cell activities through this pathway. Consistent with our previous finding that Id1 activates the Wnt/β-catenin/TCF pathway in human breast cancer [11], treatment with FH535, a small-molecule inhibitor of Wnt/β-catenin, repressed Id1-mediated c-Myc expression in MECs from MMTV-Id1 mice as confirmed by western blot and RT-PCR analysis (Figure 5C). Furthermore, inhibition of this signaling by FH535 reversed Id1-induced MaSC expansion in MECs from MMTV-Id1 mice as assessed by FACS analysis (Figure 5D). Treatment of these cells with FH535 also reduced the number of spheres and colonies in a dose-dependent manner (Figures 5E and 5F, respectively). We further determined whether c-Myc is a key molecule in the regulation of self-renewal of MaSC by Id1. Treatment of MECs from MMTV-Id1 mice with Id1 and c-Myc small interfering (si) RNAs showed that increased mammosphere formation was almost completely blocked by Id1 knockdown and partially inhibited by c-Myc depletion (Figures 5G and 5H).

**Figure 6:**
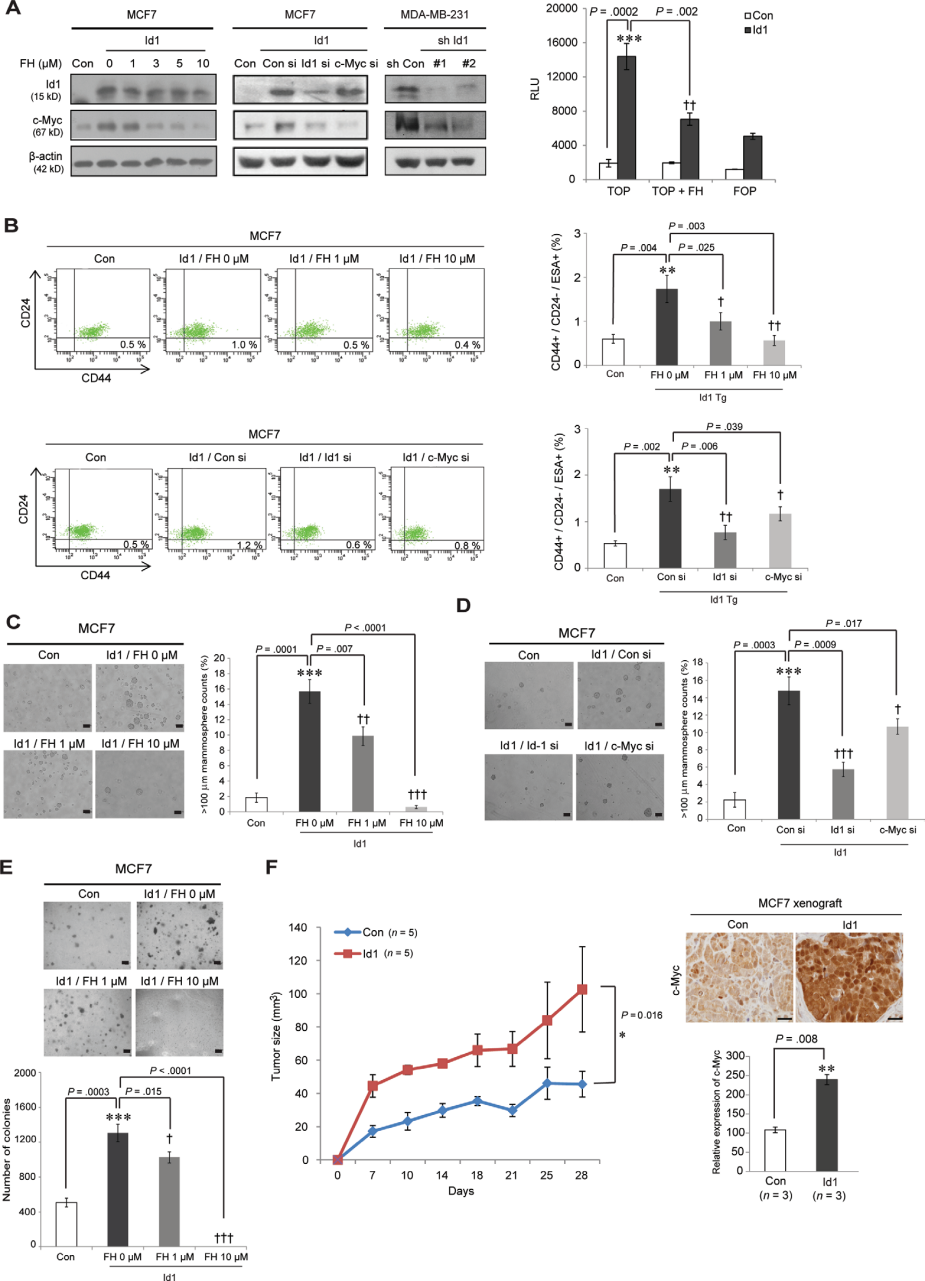
Id1 enhances breast cancer stem cell activity via Wnt/TCF signaling-mediated c-Myc regulation **A.** Cell lysates of MCF7 cells treated with FH535 or siRNA (si) against Id1 or c-Myc for 48 h and MDA-MB-231 cells expressing Id1 shRNAs were subjected to Western blot analysis using the indicated antibodies (left). Id1-overexpressing MCF7 cells transfected with TCF/LEF promoter (TOP) and its mutant form (FOP) were treated with 10 μM FH535, and the luciferase reporter activity was measured (right panel). **B.** Flow cytometry analysis of the cancer stem cell population among Id1-overexpressing MCF7 cells treated with FH535 (top panel) or transfected with Id1 or c-Myc siRNA (bottom). **C.** and **D.**, Tumorsphere-forming ability in Id1-overexpressing MCF7 cells treated with FH535 or a siRNA against Id1 or c-Myc. **E.** An anchorage-independent growth assay was performed in cells treated with FH535. Results in A–E. are shown as the means ± SD (*n* = 3 independent experiments). */^†^*P* < .05; **/^††^*P* < .01; ***/^†††^ *P* < .001 vs. Con and Id1 controls, respectively, Student *t* test). **F.** The growth curve of indicated xenograft tumors (left panel). Id1-overexpressing MCF7 cells or its control cells were orthopically injected into the nude mice. The tumor size was monitored for 28 days. Error bars represent means ± SEM (*n* = 5 independent samples; *P = 0.016, RM ANOVA). Immunohistochemistry of c-Myc in tumors derived from indicated xenograft mice (right panel). Results are shown as the means ± SD (*n* = 3 independent samples; * *P* < 0.05 vs. control, Student *t* test).

Consistent with these observations, in human breast cancer cell lines, c-Myc was upregulated by Id1 and this effect was abrogated by Id1 siRNA treatment in Id1-overexpressing MCF7 cells (Figure 6A, left panel). Similar effect was also shown in MDA-MB-231 cells expressing Id1 shRNAs. Furthermore, Id1-induced c-Myc expression and TCF/LEF promoter activity were reversed by FH535 treatment, indicating the Wnt pathway-mediated c-Myc regulation by Id1 (Figure 6A, left and right panels). Moreover, pharmacological inhibition of the Wnt/c-Myc via FH535 treatment blocked CD44^+^/CD24^−^/ESA^+^ breast CSC expansion and tumorsphere formation in Id1-overexpressing MCF7 cells (Figures 6B, top panel, 6C). Consistently, the direct inhibition of c-Myc using its siRNA showed inhibitory effect on Id1-induced CSC properties (Figures 6B, bottom panel, and 6D). The anchorage-independent tumor growth by Id1 overexpression was also abrogated by long-term treatment with FH535 in these cells (Figure 6E). These data implied that c-Myc is a downstream effector of Id1 in normal and malignant stem cells. We further confirmed that acceleration of breast tumorgenesis by Id1 (*P* = 0.016, RM ANOVA) is accompanied by increased c-Myc expression (*P* = 0.008, two-sided *t* test) in xenograft tumors (*n* = 5; Figure 6F). Collectively, these findings suggest that Wnt/TCF signaling-mediated c-Myc activation by Id1 results in oncogenic transformation of MaSCs, leading to activation of breast CSCs.

## DISCUSSION

In this study, we uncovered *in vivo* genetic evidence of Id1 function that Id1 plays an important role in regulating normal and malignant mammary stem cells in transgenic mice. MMTV-Id1 mice showed increased MaSC population and self-renewal activity. Furthermore, Id1 maintained MaSC-enriched basal cells and contributed to the generation of basal type mammary hyperplasia and tumors. Id1 also modulated both breast normal and cancer stem cells via the Wnt/TCF/c-Myc pathway. Therefore, we suggest that Id1 is critical for mammary tumorigenesis via modulation of normal and malignant mammary stem cells.

Our findings demonstrate the emerging role of Id1 in the mammary epithelial hierarchy as a determinant of mammary basal stem cell activity. Accumulating studies have shown the importance of mammary basal epithelial cells in regulation of mammary gland development and stem cell maintenance. MaSCs have characteristic features of basal cells [30]. Consistently, basal lineage cells expressing K14 contribute to the regenerative capacity in the pre-pubertal mammary glands [31, 32]. Moreover, a recent study showed that basal-to-luminal cell signaling contribute to lactogenesis [33], implying the crucial role of basal cells in functional maturation of the mammary gland. In accordance with these observations, our previous and present findings using a MMTV-Id1 transgenic mice model could suggest that the pivotal role of Id1 in mammary gland development might be due to enhanced MaSC activity and basal/myoepithelial cell lineage. Furthermore, consistent with a previous study showing that MMTV-Wnt-1 mice contained a higher number of MaSCs and high expression of basal markers [15], our data imply that the Wnt signaling is a key mediator of Id1-induced mammary gland development and MaSC maintenance. Taken together, these findings suggest that Id1/Wnt signaling cascade might have a key role in mammary gland epithelium by maintaining MaSC-enriched basal cells.

Furthermore, it is important to note that increased MaSC activity caused by Id1 in mammary glands resulted in the formation of hyperplastic nodules and breast tumors. Contrary to the previous reports suggesting that Id1 is insufficient for tumorigenesis of mammary epithelial cells [3, 34], the constitutive Id1 overexpression alone could promote breast tumorigenesis in a long period observation of our MMTV-Id1 mice, raising the possibility for emerging role of Id1 in breast tumor initiation as a MaSC regulator. For the low frequency of tumor occurrence in the MMTV-Id1 mice, we speculate that additional tumorigenic events such as Ras cooperation should be required for more facilitation of mammary tumorigenesis. Notably, our finding that Id1-induced ductal hyperplasia and breast tumors exclusively express basal markers could suggest the generation of basal-like breast cancer by Id1. The cellular origins of five defined breast cancer subtypes (luminal A, luminal B, HER2-enriched, basal-like, and claudin-low) remain to be fully identified [20]. Moreover, whether basal-like tumors associated with positive expression of basal keratins originate from basal stem cells is controversial. Based on studies to identify the origin of BRCA mutation-driven triple-negative breast cancer, the suggestion was made that basal-like tumors could arise from luminal progenitors [18, 21]. Notch signaling, which has been reported to regulate luminal progenitors, is also associated with basal-like breast tumors [19, 35, 36]. In contrast, tumors generated from MMTV-Wnt1 mice, which exhibit MaSC expansion and high expression of basal markers, showed similarities to basal-like breast cancer [15, 37–39], suggesting that Wnt signaling-mediated basal-like tumors may originate from basal stem cells. Likewise, our findings suggest that the formation of basal marker-positive tumors in MMTV-Id1 transgenic mice may be due to Id1-mediated deregulation of the MaSC-enriched basal cell population because Id1 generated the tumors without increasing luminal progenitor numbers. Moreover, our data show that basal marker-expressing breast tumors in MMTV-Id1 mice are also associated with low expression of claudins. The majority of basal-like breast cancers have been demonstrated to be triple-negative cancers, which include the claudin-low tumors, with features such as epithelial–mesenchymal transition (EMT) and a more aggressive phenotype [40]. Consistent with this, previous studies showed that high expression of Id1 was linked to EMT-related basal B breast cancer cell lines and the claudin-low tumor subtype, as well as triple-negative breast cancer [6, 9]. Thus, this evidence supports the possibility that breast tumors in MMTV-Id1 mice might be classified as the claudin-low subtype. Together, these findings could suggest that Id1 increases deregulation of mammary basal stem cells, thereby inducing basal-like breast cancer.

Our findings also demonstrate that Id1 enhances the activity of human breast CSCs as well as those of normal mammary stem cells and suggest Wnt/TCF/c-Myc signaling as a common molecular factor for linking normal and malignant mammary stem cells. Several common regulatory factors link normal and cancer stem cells. For example, abnormal activation of many normal stem cell regulators, including Notch, Hedgehog, Wnt/β-catenin, c-Myc, and Bmi-1, which promote self-renewal in various stem cells and cause neoplasia when deregulated, have been identified in the context of CSCs [41]. Notably, c-Myc appears as a crucial factor linking normal and cancer stem cells in the mammary epithelium because induction of c-Myc and other induced pluripotent stem cell (iPS) factors in non-tumorigenic MCF10A mammary epithelial cells transformed the cells into tumorigenic CD44^+^/CD24^low^ cells with CSC properties and a malignant phenotype [42]. In this study, we observed that increased stem cell population and activity by Id1 overexpression were reversed by inhibition of Wnt/TCF/c-Myc pathway in both breast normal and cancer cells. Thus, based on our results, we suggest that Wnt/TCF signaling-mediated c-Myc activation by Id1 results in oncogenic transformation of mammary stem cells, which leads to activation of breast CSCs.

In conclusion, our data provide the evidence that Id1 increases the self-renewal activity of basal stem cells in mammary glands. Ductal hyperplasia and the formation of mammary tumors positive for basal makers occurred in MMTV-Id1 transgenic mice, perhaps due to enrichment of normal and cancerous breast stem cells resulting from Wnt/c-Myc pathway activation.

## MATERIALS AND METHODS

### Mice

MMTV-Id1 transgenic mouse generation was done as previously described [13]. The procedures were approved by the institutional animal care and use committee of Hanyang University (Seoul, Korea).

### Cell culture, siRNA and drug treatment

All human breast cancer cell lines were obtained from **American Type Culture Collection** (ATCC, Manassas, VA). MCF7 cell lines stably overexpressing Id1 were generated and maintained as previously described [11]. To establish stably Id1 knockdown MDA-MB-231 cell lines, a pair of oligos encoding Id1 shRNAs (#1, sense, 5′-TGGACGAGCAGCAGGTAAACTTCAAGAGAGTT TACCTGCTGCTCGTCCTTTTTTC-3′ and antisense, 5′-TCGAGAAAAAAGGACGAGCAGCAGGTAAACT CTCTTGAAGTTTACCTGCTGCTCGTCCA-3′; #2, sense, 5′-TGCTGAAGGCCGGCAAGACATTC AAGA GATGTCTTGCCGGCCTTCAGCTTTTTTC-3′ and antisense, 5′-TCGAGAAAAAAGCTGAAGGCCG GCAA GACATCTCTTGAATGTCTTGCCGGCTTCAGCA-3′) were annealed and cloned into pLB lentiviral vector. The generation and transduction of the lentivirus particles were performed as described previously [43]. MDA-MB-231-luciferase (Luc) cells were obtained from Xenogen Corporation (Alameda, CA) and infected with lentiviruses encoding an empty vector or Id1 cDNA for orthotopic xenografts. For transient knockdown of Id1 and c-Myc, siRNAs against Id1 (Dharmacon, Chicago, IL) and c-Myc (Bioneer, Daejon, Korea) were transfected into the cells with Lipofectamine 2000 (Invitrogen, Carlsbad, CA) under 1% serum conditions for 48 h. An inhibitor of the β-catenin/TCF pathway, FH535, was purchased from Calbiochem (Nottingham, UK).

### Antibodies

Antibodies specific for c-Myc (Cell Signaling Technology, Beverly, MA) and β-actin (Sigma, St. Louis, MO) were used for Western blot analysis. For immunostaining analysis, antibodies specific for the following proteins were used: ER, PR, SMA, and K5 (Novus Biologicals, Littleton, CO); K14, vimentin, and p63 (Bioworld Technology, Louis Park, MN); K8 (Abcam, Cambridge, MA); c-Myc (9E10) and HA probe (Santa Cruz Biotechnology, Santa Cruz, CA); claudin 3 and claudin 4 (Invitrogen). An antibody specific for Id1 (C-20, Santa Cruz Biotechnology) were used for both Western blot and immunostaining analysis.

### Isolation of mouse primary MEC and MaSCs

Primary mammary epithelial cells (MECs) were prepared from 12-week-old FVB mice as previously described [13]. To separate MaSCs from the purified mouse MECs, cells were stained with CD24-FITC (BD Biosciences, San Jose, CA) and CD49f-APC (BioLegend, San Diego, CA), and CD24^med^/CD49f^high^ cells was then collected using a FACSAria flow cytometer (BD Biosciences).

### Flow cytometry analysis

To analyze mammary stem and progenitor populations, Lin^−^ cells isolated from mouse MECs were incubated with CD24-FITC, CD49f-APC, and/or CD61-FITC antibodies (BD Biosciences) on ice for 15 min. To measure the breast CSC population, cells were labeled with ESA-FITC, CD44-APC, and CD24-PE antibodies (BD Biosciences) at 4°C for 15 min. The analysis was performed using a FACSCanto Flow Cytometer (BD Biosciences). To measure basal and luminal marker expression, cells were stained with primary antibodies against basal and luminal markers, incubated with goat anti-mouse Cy5 (Invitrogen) for luminal markers or goat anti-rabbit Alexa Fluor 488 (Invitrogen) for basal markers, and analyzed by flow cytometry.

### Sphere formation and anchorage-independent growth assays

Freshly isolated mouse MECs were plated on 6-well ultralow attachment plates (Costar, Corning, NY) in DMEM-F12 GlutaMAX medium (Invitrogen) supplemented with B27 (Invitrogen), 10 ng/mL epidermal growth factor (R&D Systems, Minneapolis, MN), 10 ng/mL basic fibroblast growth factor (PeproTech, Rocky Hill, NJ), and 4 μg/mL heparin (Sigma). The serial sphere forming assays was then examined as described previously [43]. For soft agar colony-forming assay, 1 × 10^5^ cells (for MCF7 and MDA-MB-231 cells) or 1 × 10^3^ cells (for MECs) suspended in 0.3% noble agar solution dissolved in primary culture medium were added to the bottom agar layer, and the formation of colonies (>50 μm) was scored using a light microscope after 4 weeks, as described previously [43].

### Immunohistochemistry and Immunocytochemistry

Immunohistochemical analysis was performed as described previously [13]. Immunocytochemistry analysis of MECs, MaSCs, and MaCFCs were performed following the manufacturer's instructions of Immunodetection Kit (Vector, Burlingame, CA). A fluorescence microscope (Nikon, Tokyo, Japan) was used for detecting immunofluorescence.

### *In vitro* epithelial colony-forming assay

To characterize bipotent mammary epithelial progenitor cells as described previously [23, 24, 44], single-cell suspensions obtained by dissociation of primary mammospheres were seeded on a feeder layer of irradiated NIH 3T3 cells for 72 h. The mammary colonies were then fixed, stained with crystal violet, and three distinct types of colonies (luminal colonies with tightly arranged cuboidal cells, myoepithelial colonies with dispersed teardrop shape, and mixed colonies with combined luminal and myoepithelial shapes) were counted under a microscope.

### 3-D differentiation culture assay

Single cells obtained by dissociation of primary mammosphere were embedded in Matrigel on 24-well plates and incubated in EpiCult-B medium (Stem Cell Technologies, Vancouver, Canada) containing 1% fetal bovine serum. After 3 weeks, the colony structures generated from Matrigel were counted under a microscope.

### Western blot analysis

Cells were lysed in radioimmunoprecipitation assay (RIPA) buffer supplemented with protease inhibitors (Roche Applied Science, Indianapolis, IN) and phosphatase inhibitor cocktails (Sigma). Western blot analysis was then performed as previously described [13, 45].

### RT-PCR

Total RNA was isolated from MECs and MaSCs using TRIzol reagent (Invitrogen) and an RNeasy Micro Kit (Qiagen, Valencia, CA), respectively, as previously described [13]. cDNA was synthesized from 1 μg of total RNA using M-MLV reverse transcriptase (Promega, Madison, WI, USA) according to the manufacturer's protocol. Primers with the following sequences were used for PCR: mouse *c-Myc*, forward primer 5′-CGC GATCAGCTCTCCTGAAA-3′ and reverse primer 5′-CTA ACCGGCCGCTACATTCA-3′; mouse *Id1*, forward primer 5′-TCTGTCGGAGCAAAGCGTGGCC-3′ and reverse primer, 5′-CCGGTGGTCCCGACTTCAGACT; and mouse *18s*, forward primer 5′-GTAACC CGTTGAACCCCATT-3′ and reverse primer 5′-CCATC CAATCGGTAGTAGCG-3′.

### Luciferase reporter assay

To analyze the transcriptional activity of the Wnt/β-catenin/TCF pathway, cells were co-transfected with pTOP-FLASH, a TCF/LEF promoter, or pFOP-FLASH, a TOP mutant form, and β-galactosidase cDNA by Lipofectamine 2000 (Invitrogen). After 24 h transfection, cells were treated with FH535 for 24 h. Then, the luciferase reporter activity from the cell lysates were measured using a MicroLumat Plus LB96V luminometer (Berthold Technologies, Bad Wildbad, Germany). The luciferase activity, expressed as relative light units (RLUs), was normalized to β-galactosidase activity.

### Mouse stem cell PCR arrays

The expression levels of mouse stem cell-associated genes were examined using Mouse Stem Cell RT^2^ Profiler PCR Arrays (SABiosciences, Frederick, MD) and validated with Real-time PCR using SABiosciences RT^2^ qPCR Master Mix on a 7300 Real-Time PCR System (Applied Biosystems, Foster City, CA) in accordance with the manufacturer's instructions. The relative expression values of genes were normalized to housekeeping gene expression levels. Samples were analyzed in triplicate and data were analyzed at the company Web site (http://pcrdataanalysis.sabiosciences.com/pcr/array-analysis.php). Pathway analysis of target gene lists was conducted using DAVID bioinformatics resources (david.abcc.ncifcrf.gov/).

### Mammary cell transplantation assay

MECs and MaSCs isolated from mice were injected into the cleared fat pads of 21-day-old female FVB mice. After 8 weeks, the fat pads were excised and stained with carmine alum. Then, the percentage of fat pads filled and the positive outgrowth fraction were determined as previously described [46, 47].

### Whole-mount analysis of mammary glands

Recipient glands were dissected, spread on glass slides, and fixed in Carnoy's solution (ethanol/chloroform/glacial acetic acid in a 6:3:1 ratio) for 24 h. The fixed glands were washed in 70% ethanol for 15 min, rinsed with water for 5 min, and stained with carmine alum for 24 h. The stained tissues were dehydrated, cleared using a graded ethanol series and xylene, and mounted with Permount. After photography of whole mammary glands, the tissues were embedded in paraffin. Sections (4 μm) were rehydrated and stained with hematoxylin and eosin (H&E).

### Human breast cancer orthotopic xenograft assay

Five-week-old female nonobese diabetic (NOD)/SCID mice were purchased from the Korea Research Institute of Bioscience & Biotechnology (KRIBB, Daejeon, Korea) and kept under specific pathogen-free conditions. MDA-MB-231-Luc cells infected with lentiviruses encoding an empty vector or Id1 cDNA (1 × 10^6^ cells) suspended in Matrigel (BD Biosciences) were injected into the mammary fat pads on the left and right sides, respectively, of the same animal. One week after injection, mice were given a solution of D-luciferin (150 mg/kg in PBS; Caliper Life Sciences, Hopkinton, MA) and anesthetized with 1% isoflurane/oxygen. The imaging and quantification of light emitted from the bioluminescent tumors were conducted using the IVIS camera system (Xenogen Corporation) and Living Image analysis software (Xenogen Corporation). Tumor volume was calculated using as 1/2 × long diameter × short diameter^2^. Tumor growth was measured twice weekly. For xenografts with MCF7 cells, BALB/c-nude mice were supplemented with estradiol pellets (0.72 mg/pellet, 60 day release; Innovative Research of America, Sarasota, FL) 1 week before cell implantation. The stable Id1-overexpressing MCF7 cells or control cells (2 × 10^6^ cells) were suspended in Matrigel and then injected into the mammary fat pads of the nude mice. Tumor volume was monitored as described above for 28 days.

### Statistical analyses

Statistical significance of the differences between controls and experimental groups was determined with the two-sided, unpaired Student *t* test using Excel spreadsheets (Microsoft, Redmond, WA) and SPSS software (version 12.0; SPSS Inc., Chicago, IL). To estimate the frequency of long-term regenerative MaSCs and the tumor-initiating ability of CSCs, limiting dilution transplantation results were analyzed using L-Calc software [46, 47], and statistical significance of the data was calculated by Poisson distribution using SAS software. The percentage differences of reconstituted ductal outgrowth and the incidence rate of hyperplasia/tumor in each group of mice were determined by Mann-Whitney *U* test and chi-square test, respectively, using SPSS software. For comparison of cell growth rates between different groups in xenograft, repeated measures analysis of variance (RM ANOVA) was used. *P* values less than 0.05 were considered statistically significant.

## SUPPLEMENTARY FIGURE AND TABLE


