# Supplementary material for: Overexpression of Id1 in transgenic mice promotes mammary basal stem cell activity and breast tumorigenesis
Source: Oncotarget. 2015 Apr 16;6(19):17276–90. doi: 10.18632/oncotarget.3640 (PMC4627307; doi:10.18632/oncotarget.3640)
Supplement: Supplementary file 1 [file oncotarget-06-17276-s001.pdf]

SUPPLEMENTARY FIGURE AND TABLE

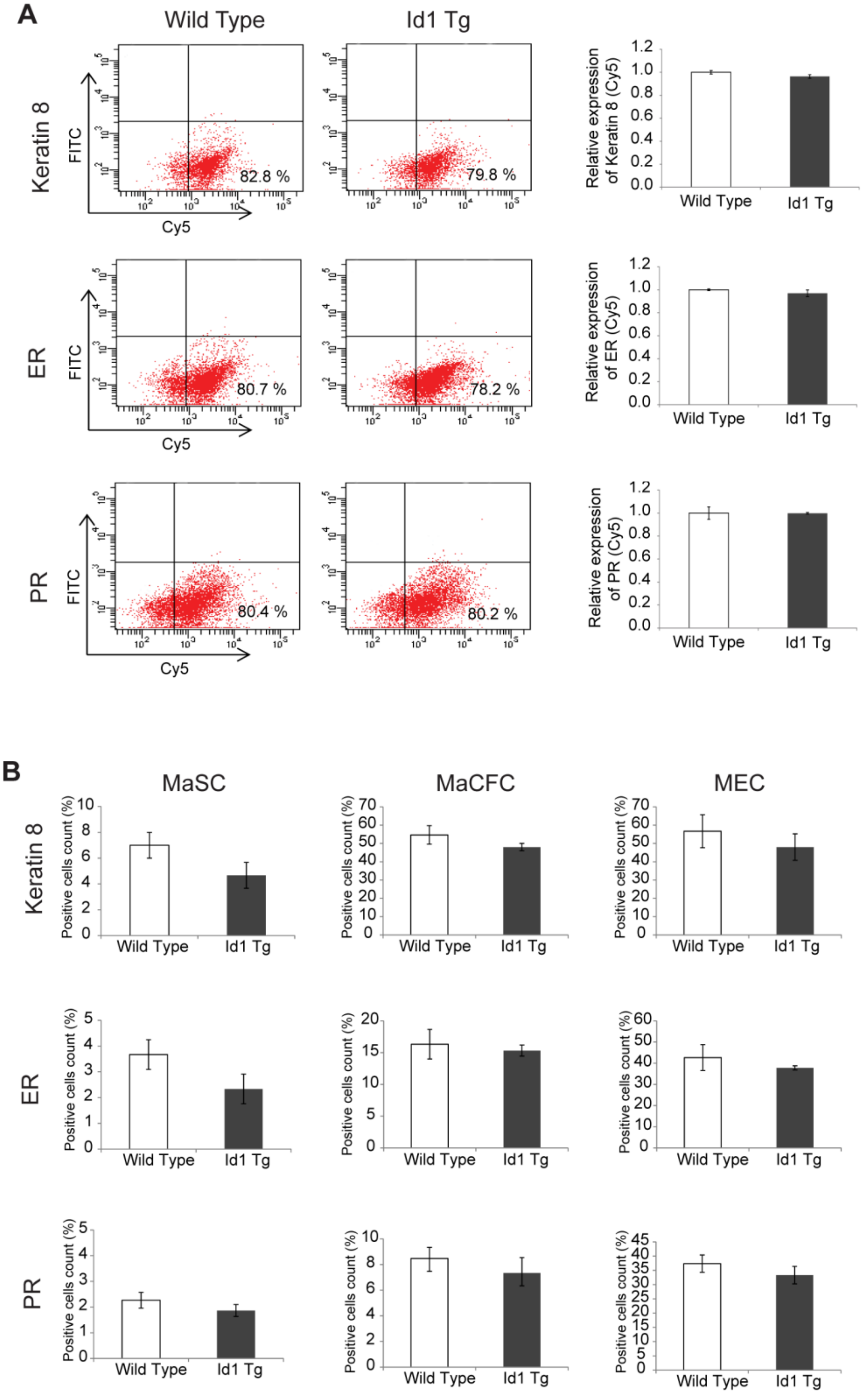

Supplementary Figure S1: Expression of luminal markers in MMTV-Id1 transgenic mice. A. Flow cytometry analysis of MECs derived from virgin wild-type and MMTV-Id-1 transgenic mice using antibodies against the luminal markers keratin 8, ER, and PR. The dot plot shows the percentage of luminal marker-positive cells (Cy5-positive; lower right quadrant). The FITC channel was used as a negative control to determine background fluorescence. **B.** Quantitative immunocytochemical analysis results of the expression of the luminal markers keratin 8, ER, and PR in MaSCs, Ma-CFCs, and MECs (shown as a bar graph). The results of all experiments are shown as the means ± SD (n = 3 independent experiments; *P < 0.05 vs. control, Student t test).

Supplementary Table S1: Effect of Id1 on the expression of mouse stem cell-specific genes

Gene symbol	Description	Fold change	*p*-value
Upregulated genes			
Cd3d	CD3 antigen, delta polypeptide	8.99	0.022
T	Brachyury	5.74	0.034
Ncam1	Neural cell adhesion molecule 1	3.34	0.024
Dhh	Desert hedgehog	3.33	0.006
Actc1	Actin, alpha, cardiac muscle 1	3.24	0.014
Hdac1	Histone deacetylase 1	3.09	0.018
Rb1	Retinoblastoma 1	2.94	0.047
Cd8b1	CD8 antigen, beta chain 1	2.41	0.008
Cd44	CD44 antigen	2.15	0.008
Cdh2	Cadherin 2	1.94	0.000
Wnt1	Wingless-related MMTV integration site 1	1.94	0.040
Cd4	CD4 antigen	1.91	0.022
Myc	Myelocytomatosis oncogene	1.89	0.000
Fgf1	Fibroblast growth factor 1	1.76	0.033
Ctnna1	Catenin (cadherin-associated protein), alpha 1	1.75	0.034
Ccnd2	Cyclin D2	1.72	0.021
Apc	Adenomatosis polyposis coli	1.72	0.034
Msx1	Homeobox, msh-like 1	1.57	0.030
Fgfr1	Fibroblast growth factor receptor 1	1.53	0.029
Ccna2	Cyclin A2	1.52	0.020
Gdf2	Growth differentiation factor 2	1.50	0.001
Downregulated genes			
Aldh2	Aldehyde dehydrogenase 2, mitochondrial	–10.64	0.040
Frat1	Frequently rearranged in advanced T-cell lymphomas	–6.64	0.018
Notch1	Notch gene homolog 1 (*Drosophila*)	–3.20	0.046
Btrc	Beta-transducin repeat-containing protein	–2.90	0.020
Dll1	Delta-like 1 (*Drosophila*)	–2.87	0.038
Tert	Telomerase reverse transcriptase	–2.53	0.022
Ep300	E1A binding protein p300	–2.25	0.020
Myst2	MYST histone acetyltransferase 2	–2.22	0.009
Fzd1	Frizzled homolog 1 (*Drosophila*)	–1.92	0.003
Axin1	Axin 1	–1.78	0.000
Mme	Membrane metallo-endopeptidase	–1.54	0.000

Relative expression of mouse stem cell genes in MaSCs from MMTV-Id1 transgenic mice compared to those from wild-type mice was analyzed using Mouse Stem Cell RT^2 Profiler™ PCR Arrays. Samples were examined in triplicate and data were analyzed statistically by Student's *t* test.
